# Nucleolar Integrity Is Required for the Maintenance of Long-Term Synaptic Plasticity

**DOI:** 10.1371/journal.pone.0104364

**Published:** 2014-08-04

**Authors:** Kim D. Allen, Andrei V. Gourov, Christopher Harte, Peng Gao, Clarice Lee, Darlene Sylvain, Joshua M. Splett, William C. Oxberry, Paula S. van de Nes, Matthew J. Troy-Regier, Jason Wolk, Juan M. Alarcon, A. Iván Hernández

**Affiliations:** 1 Department of Pathology, State University of New York, Downstate Medical Center, Brooklyn, New York, United States of America; 2 Department of Biology, School of Science, Health and Technology, City University of New York, Medgar Evers College, Brooklyn, New York, United States of America; 3 The Robert F. Furchgott Center for Neural and Behavioral Science, State University of New York, Downstate Medical Center, Brooklyn, New York, United States of America; 4 Departments of Physiology and Pharmacology, State University of New York, Downstate Medical Center, Brooklyn, New York, United States of America; University of Louisville, United States of America

## Abstract

Long-term memory (LTM) formation requires new protein synthesis and new gene expression. Based on our work in *Aplysia*, we hypothesized that the rRNA genes, stimulation-dependent targets of the enzyme Poly(ADP-ribose) polymerase-1 (PARP-1), are primary effectors of the activity-dependent changes in synaptic function that maintain synaptic plasticity and memory. Using electrophysiology, immunohistochemistry, pharmacology and molecular biology techniques, we show here, for the first time, that the maintenance of forskolin-induced late-phase long-term potentiation (L-LTP) in mouse hippocampal slices requires nucleolar integrity and the expression of new rRNAs. The activity-dependent upregulation of rRNA, as well as L-LTP expression, are poly(ADP-ribosyl)ation (PAR) dependent and accompanied by an increase in nuclear PARP-1 and Poly(ADP) ribose molecules (pADPr) after forskolin stimulation. The upregulation of PARP-1 and pADPr is regulated by Protein kinase A (PKA) and extracellular signal-regulated kinase (ERK)—two kinases strongly associated with long-term plasticity and learning and memory. Selective inhibition of RNA Polymerase I (Pol I), responsible for the synthesis of precursor rRNA, results in the segmentation of nucleoli, the exclusion of PARP-1 from functional nucleolar compartments and disrupted L-LTP maintenance. Taken as a whole, these results suggest that new rRNAs (28S, 18S, and 5.8S ribosomal components)—hence, new ribosomes and nucleoli integrity—are required for the maintenance of long-term synaptic plasticity. This provides a mechanistic link between stimulation-dependent gene expression and the new protein synthesis known to be required for memory consolidation.

## Introduction

More than half a century has passed since it was first noted that LTM formation requires new protein synthesis [1] and new gene expression [2] (reviewed by [3])—ideas that transformed the field of learning and memory by propelling it into the arena of molecular genetics. Additionally, persistent forms of synaptic plasticity such as long-term facilitation (LTF) in invertebrates and long-term potentiation (LTP) in mammals have provided valuable models for investigating the molecular mechanism that underlies memory formation, consolidation and maintenance [3], [4].

Significant progress has been made toward understanding the molecular events that initiate the translational and genomic response to synaptic stimulation [5]–[7]. For reviews see [8], [9]. Still, relatively little is known about the mechanism whereby activity-induced transcription is coupled to selective translation in order to perpetuate synapse specific changes.

Most efforts to understand experience-induced changes in neuronal gene expression have focused on the transcription products of RNA polymerase II (precursor mRNA, snRNA and microRNA). Yet, the identities of the gene products that are both necessary and sufficient to consolidate activity-dependent, long-term plastic changes have remained elusive.

We and others have shown that the chromatin-remodeling enzyme, PARP-1, is an important epigenetic regulator of gene expression required for synaptic plasticity and memory in invertebrates and mammals [10]–[13]. In *Aplysia*, we found that PARP-1 is necessary for LTF, and that among the activity-dependent genes upregulated by PARP-1 were ribosomal RNA genes (rDNA) [11]. Transcription of rDNA is carried out by RNA polymerase I (Pol I) in the nucleolus, a dynamic intranuclear organelle where rRNA synthesis, processing, and ribosome assembly take place. PARP-1 is abundant in the nucleolus and has been shown to be essential for the inheritance of rDNA chromatin organization [14], as well as the processing of pre-rRNAs and their assembly into ribosomes [15]. Based on these findings, we hypothesized that crucial among the new gene products required for LTP are new rRNA components of the translational machinery itself, and that this activity-dependent gene expression is modulated by PARP-1. Here we report that nucleolar integrity and new rRNA gene expression are required for the maintenance of LTP in mouse hippocampal slices, and that this inducible transcriptional activity of Pol I is regulated by PKA/ERK-dependent PAR.

## Materials and Methods

### Drugs

**Figure 1:**
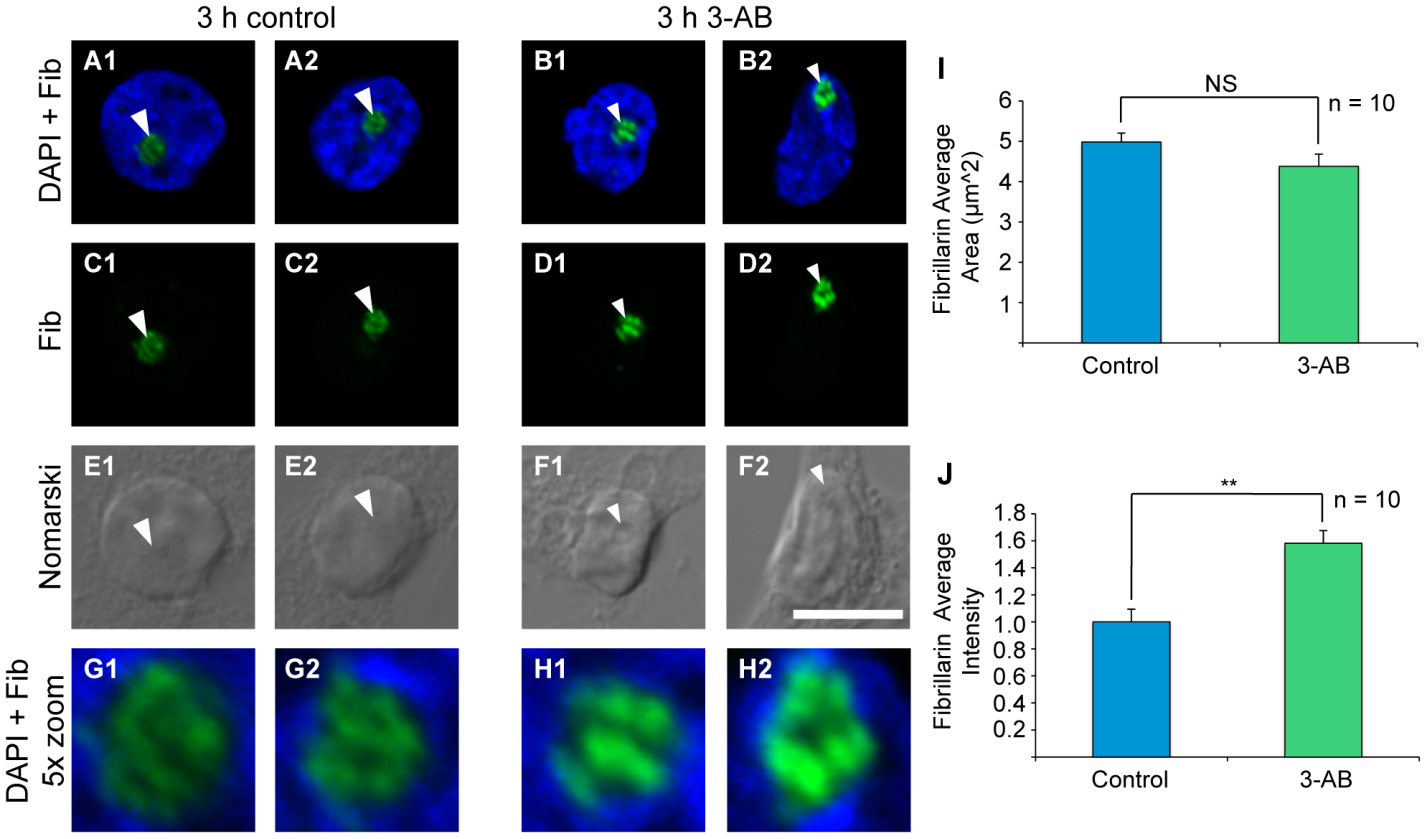
PAR inhibitor 3-AB produces changes in nucleolar fibrillarin intensity in cultured hippocampal neurons. DAPI staining (blue; A, B, G and H) shows the area of nuclei. Nomarski images (grey; E and F) show the area of the nuclei and nucleoli (arrowheads). (I) 3-AB treatment (100 µM, 3h) has no effect on the average area of the nucleolar marker fibrillarin (green). (J) In contrast, there was an increase in the average intensity of fibrillarin that was ∼1.6 fold stronger than control; compare arrowheads A,C,G to B, D,H. Bar = 10 µm (A–F) and 2 µm (G, H). Student's t-test ** = p<0.01; NS = not significant.

**Figure 2:**
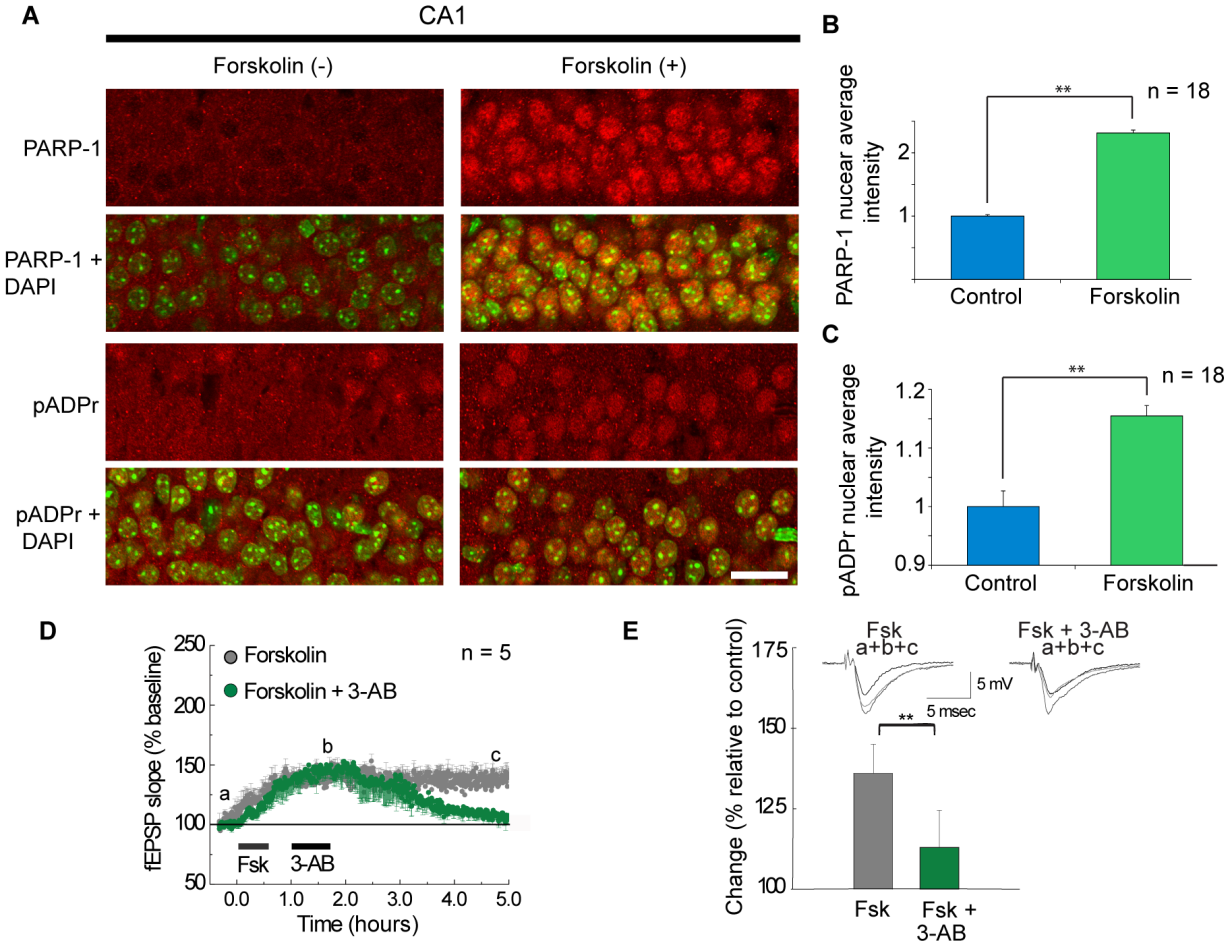
Forskolin (Fsk) induces an increase in nuclear PARP-1 and pADPr; PAR is required for the maintenance of LTP. (A) PARP-1 (top two rows) and pADPr (bottom two rows) are increased after Fsk treatment in hippocampal slices (compare left columns to right columns in A). Top row: PARP-1 staining (red) of control (Left) and Fsk treated (50 µM, 30 min) slices (Right). Second row: Merged images of PARP-1 and DAPI nuclear counterstaining (green). Third row: pADPr staining (red) of control (Left) and Fsk treated slices (Right). Bottom row: Merged images of pADPr and DAPI; Bar in A = 10 µm. (B) Fsk treatment produces a 2.3 fold increase of nuclear PARP-1 and (C), a 1.2 fold increase of nuclear pADPr. (D) Brief 45 min application of PAR inhibitor 3-AB (100 µM) disrupts LTP in the CA1 region of mouse hippocampal slices. (E) Bars represent the quantification of the change in LTP amplitude 4 h after initiating treatment. Representative fEPSP traces for times a, b and c (from D) appear above bars. Student's t-test (B and C). Student-Newman-Keuls test (E); ** = p<0.01.

**Figure 3:**
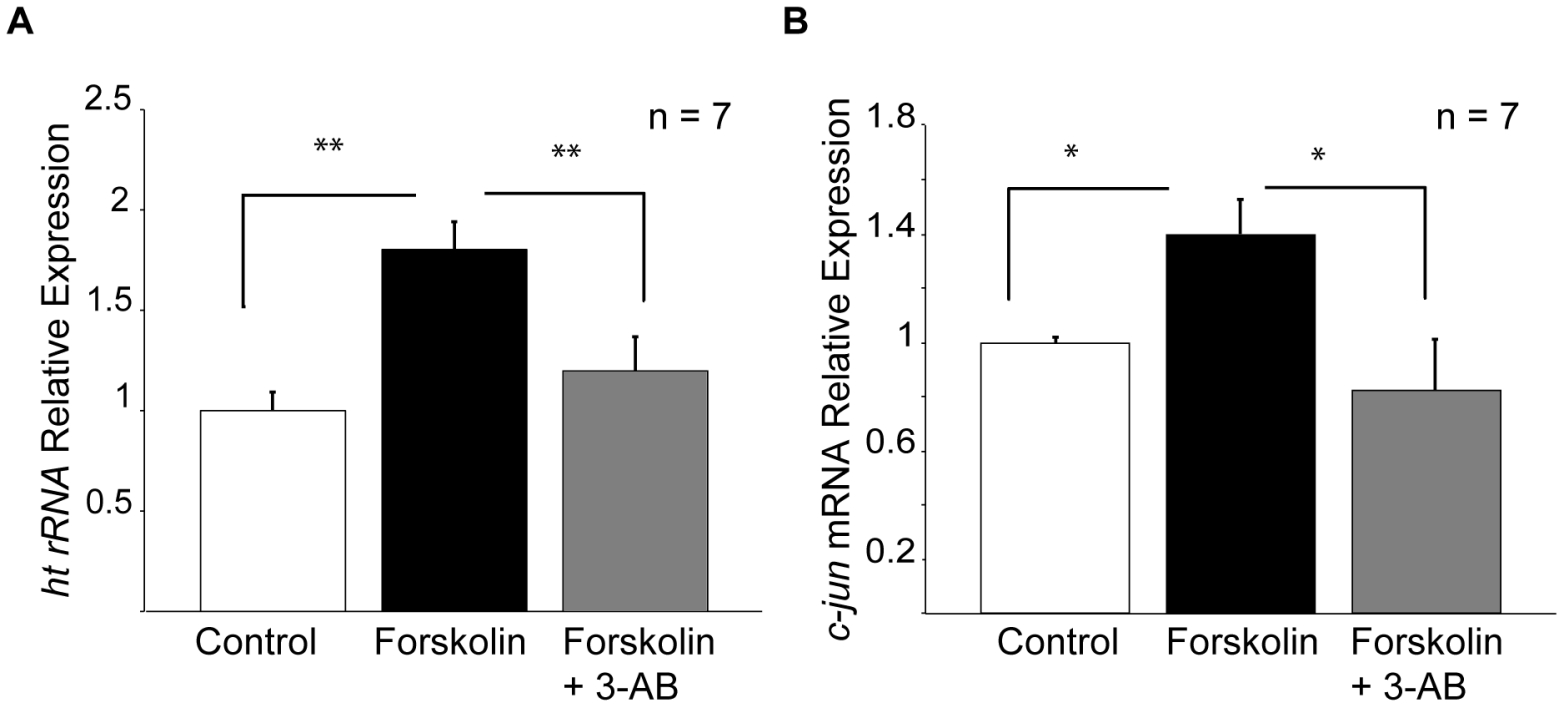
PAR is required for Fsk-induced synthesis of new rRNAs. (A) Fsk treatment upregulates unedited precursor rRNA (ht rRNA) (black bar) 1.8 fold in hippocampal slices compared with untreated controls (white bar). This increase is prevented when PAR is blocked by 3-AB (grey bar). (B) The PARP-1 dependent immediate-early gene *c-jun*, was used as a positive control. Student's t-test; * = p<0.05; ** = p<0.01.

**Figure 4:**
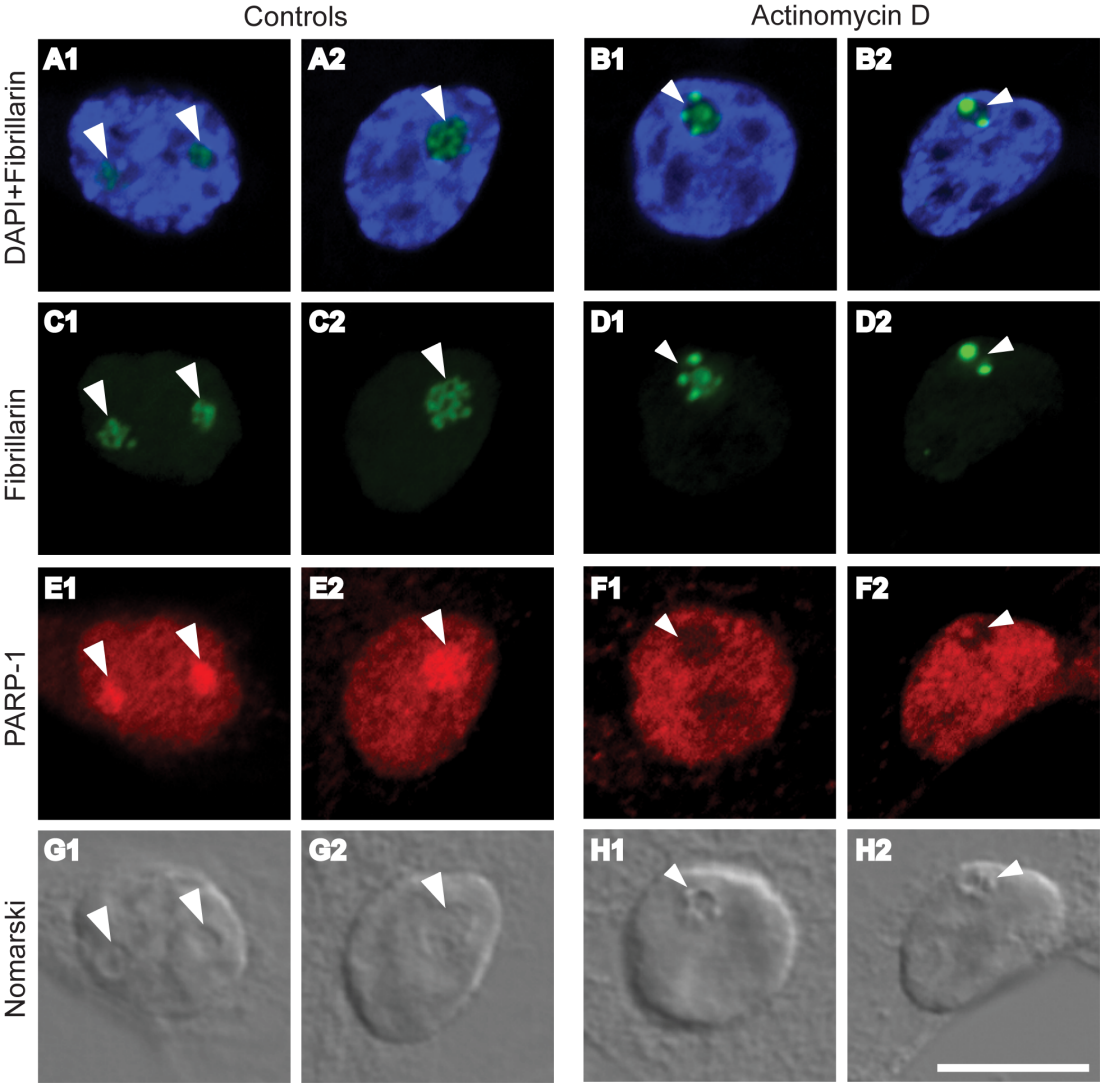
Low-dose Actinomycin D (Act-D) treatment dramatically disrupts nucleolar localization of PARP-1 in cultured hippocampal neurons. DAPI staining (blue; A, B) shows the area of the nucleus. Nomarski images (grey; G and H) show the area of the nuclei and nucleoli (shown by arrowheads). Low-dose Act-D treatment (100 nM, 45 min) causes nucleolar disruption as indicated by the distribution of fibrillarin (green). Fibrillarin forms punctate domains of increased intensity under Act-D treatment (compare large arrowheads A,C to small arrowheads B,D). Under the same treatment PARP-1 (red) exits the nucleolus (compare large arrowheads in E to small arrowheads in F). Bar = 10 µm.

**Figure 5:**
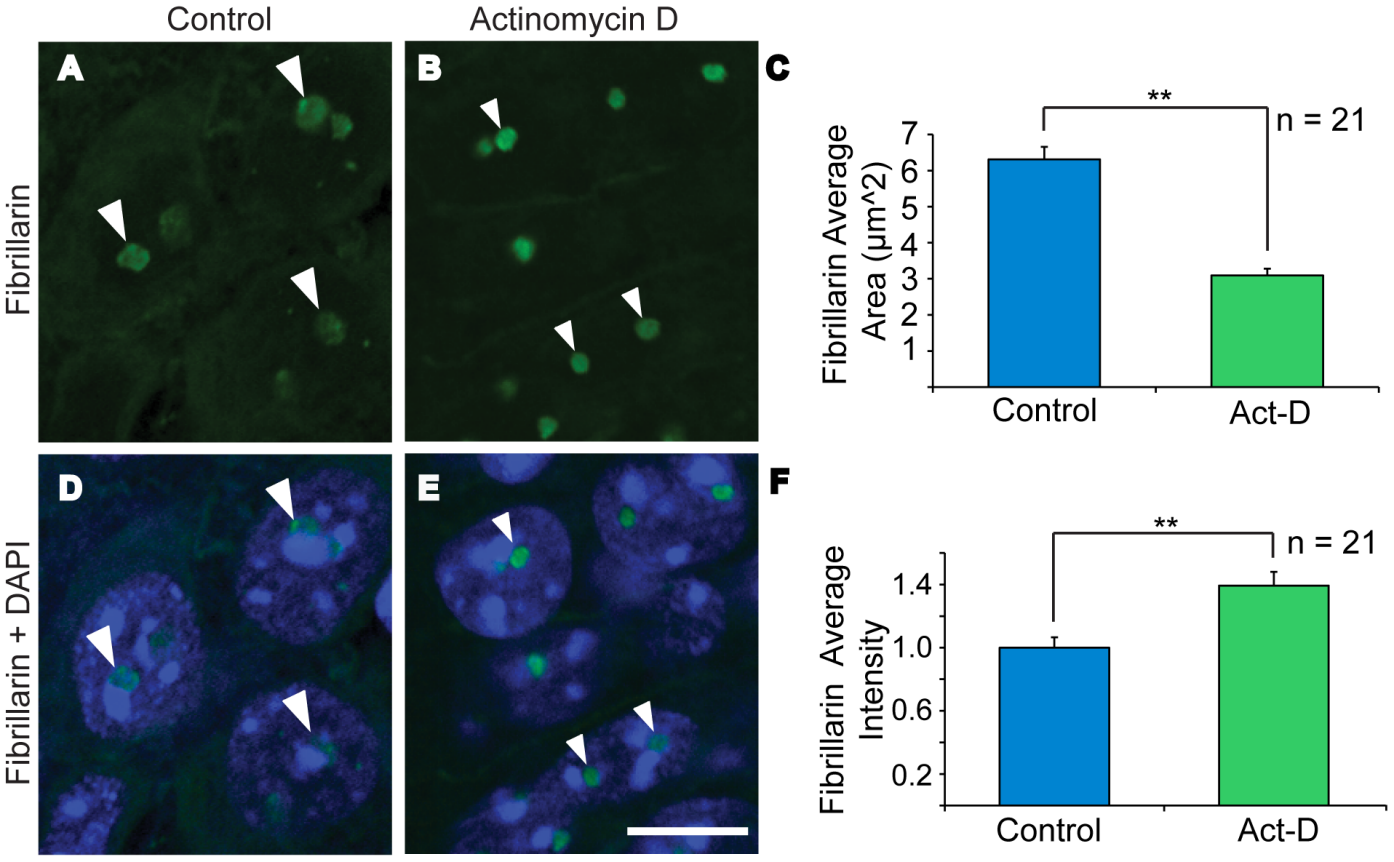
Low-dose Act-D treatment causes nucleolar disruption in mouse hippocampal slices. (A, B) Fibrillarin staining (green). (D, E) Fibrillarin+DAPI nuclear counterstaining (blue) merged. (C) Average area of fibrillarin. (F) Average intensity of fibrillarin. Left image column (A, D): Control slices show cellular distribution of fibrillarin within nucleoli (large arrowheads) with an average area of 6.3 µm^2^ (C). Right image column (B, E): Under Act-D treatment (100 nM, 45 min) fibrillarin forms punctate domains (small arrowheads, B and E) with an average area of only 3.1 µm^2^ (C) and intensity that is 1.4 times greater than control (F). Image bar = 10 µm; Student's t-test (C,F) ** = p<0.01.

**Figure 6:**
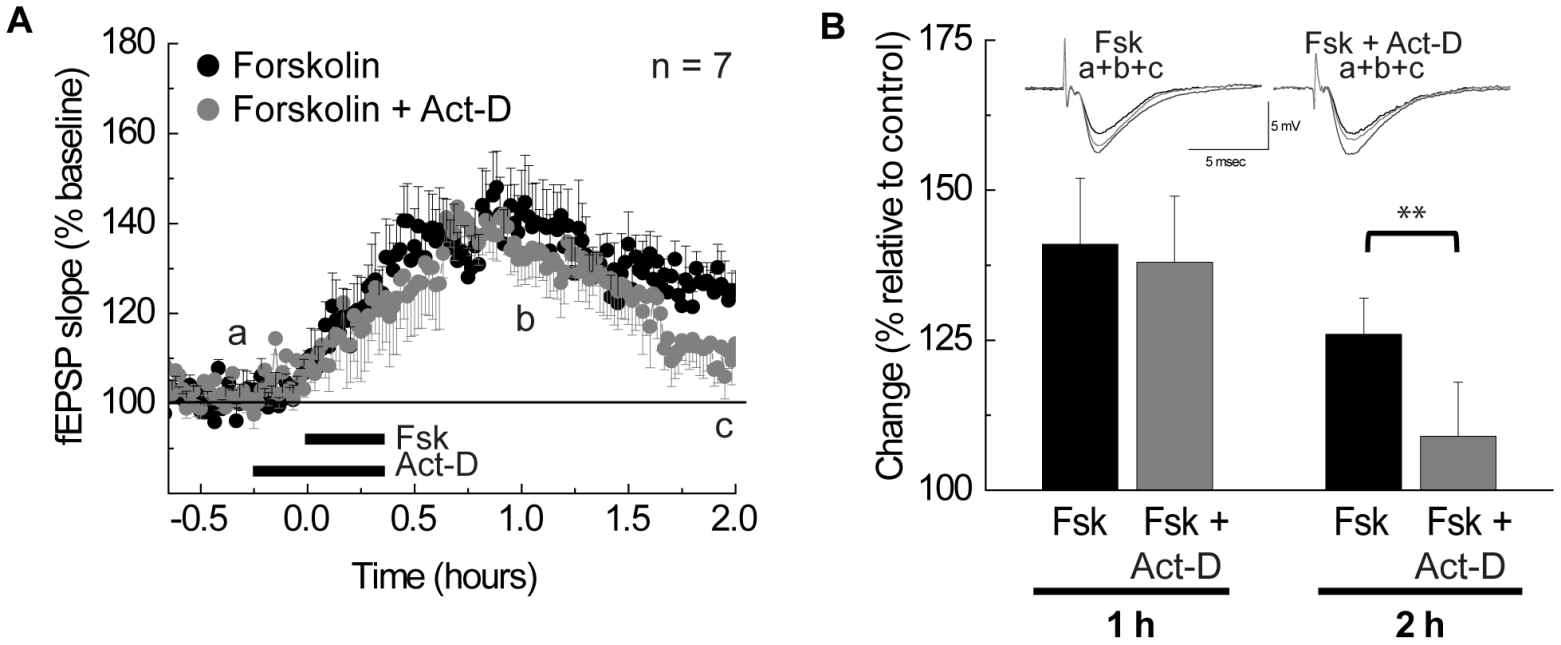
Low-dose Act-D treatment blocks LTP maintenance. (A) The maintenance of LTP is blocked by low-dose (45 min, 100 nM) Act-D treatment. (B) Quantification of the change in LTP amplitude 1 and 2 h after initiating Fsk treatment. Representative fEPSP traces for times a, b, and c (from A) appear above bars. Student-Newman-Keuls test ** = p<0.01.

**Figure 7:**
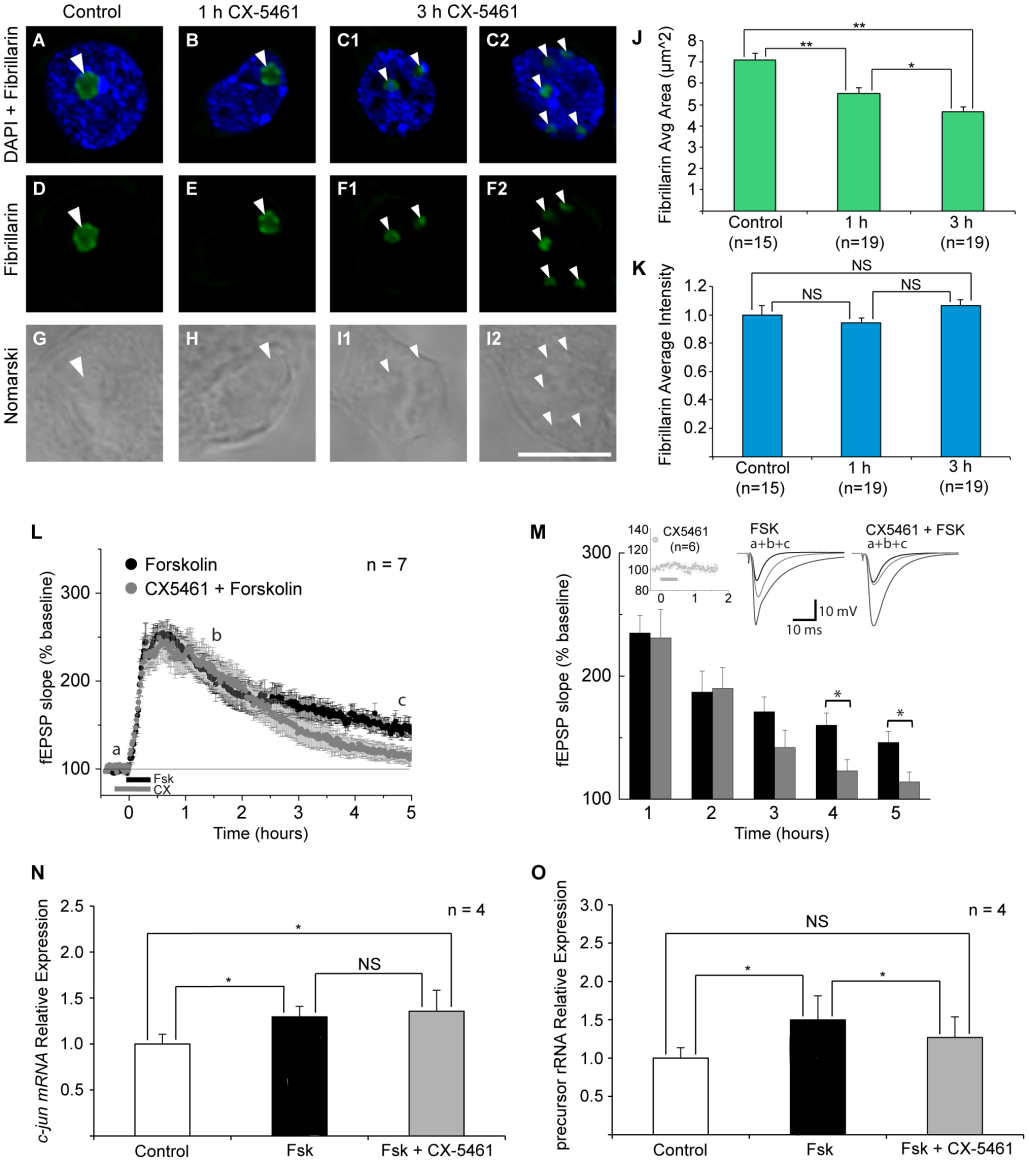
The specific Pol I inhibitor CX-5461 causes nucleolar disruption, blocks LTP maintenance and Fsk-induced synthesis of new rRNA. DAPI staining (blue; A, B,C) shows the area of the nucleus. Nomarski images (grey; G, H,I) show the area of the nuclei and nucleoli (arrowheads). Application of Pol I specific inhibitor CX-5461 (200 nM) causes nucleolar disruption as indicated by the distribution of fibrillarin (green); compare A, D to B, E and C, F. (J) After 1 h CX-5461 treatment, the average area of fibrillarin staining became smaller than control (5.5 µm^2^ and 7.1 µm^2^ respectively). After 3 h of CX-5461, fibrillarin formed small punctate domains indicative of nucleolar disruption (average area 4.7 µm^2^). (K) No changes in the average intensity of fibrillarin staining were observed after CX-5461 application. (L) The maintenance of LTP is blocked by application of CX-5461 (200 nM) (45 min). (M) Quantification of the changes in LTP amplitude between Fsk and Fsk+CX-5461 groups show significant changes 4 and 5 h after initiating Fsk treatment. CX-5461 alone had no effect on baseline stimulation (left inset). Representative fEPSP traces for times designated a, b and c in (L) are shown in middle and right inset. N) Fsk treatment upregulates the immediate-early gene *c-jun* (black bar) in hippocampal slices compared with untreated controls (white bar). This increase is not affected by Pol I inhibitor CX-5461 (grey bar). O) Fsk treatment upregulates unedited precursor rRNA (ht rRNA) (black bar) in hippocampal slices compared with untreated controls (white bar). This increase is prevented when Pol I inhibitor CX-5461 is applied during Fsk treatment (grey bar). Student's t-test (J,K,N,O). Student-Newman-Keuls test (M). * = p<0.05; ** = p<0.01; Bar = 10 µm; NS = not significant.

**Figure 8:**
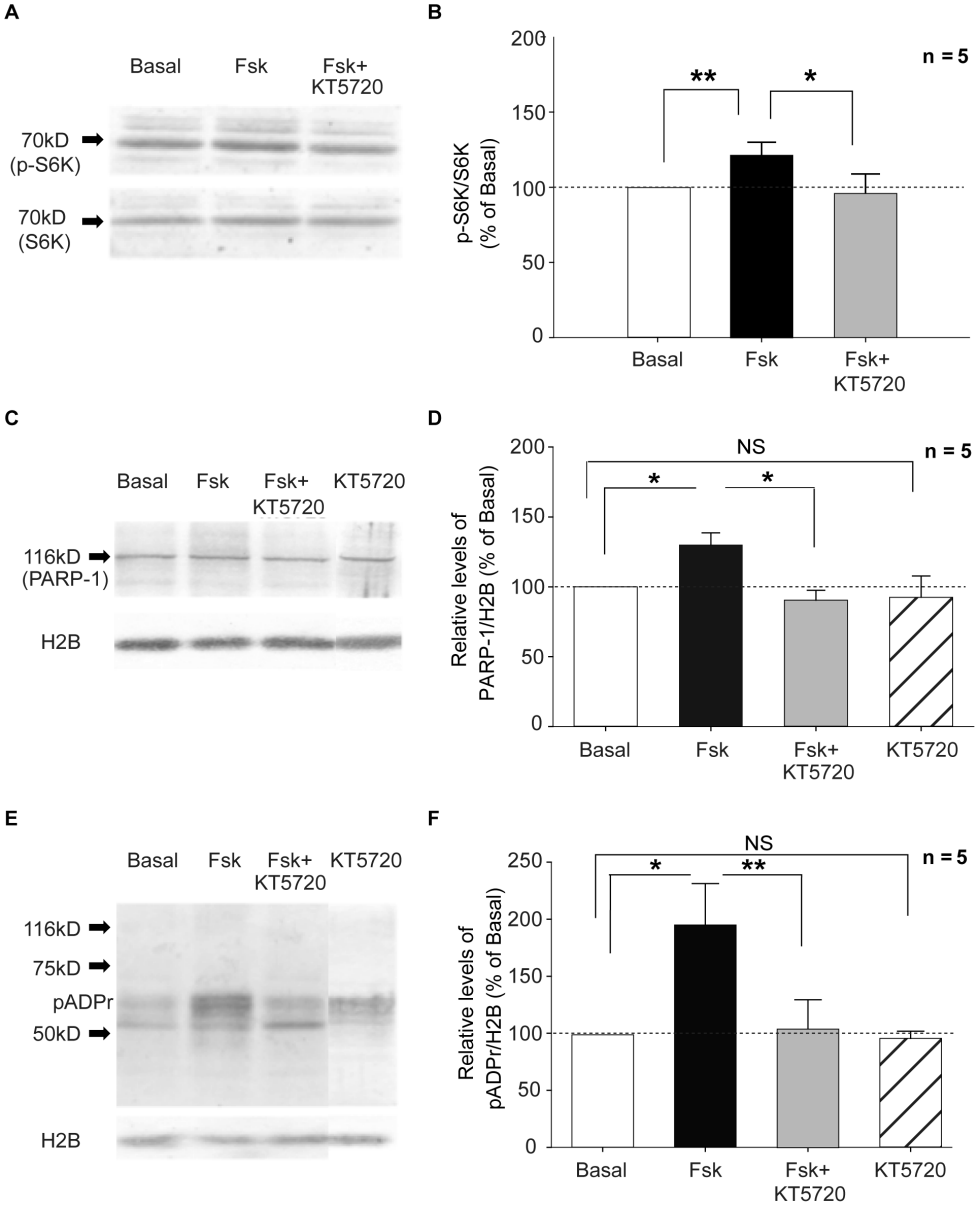
Forskolin-induced PARP-1 and pADPr increase requires PKA activity. Left column (A, C, E): Representative Western blots used for quantification shown in right column (B, D, F). Phosphorylation of the PKA pathway substrate S6K was used as a stimulation-dependent positive control quantified as the ratio of p-S6K to total S6K (A, B). Histone 2B was used as a loading control (C, E). Fsk treatment (black bar) produced an increase of p-S6K, PARP-1, and pADPr (B, D, and F, respectively). The Fsk induced upregulation of both PARP-1 and pADPr was prevented by the PKA inhibitor KT5720 (grey bar; B, D, F). The inhibitor alone had no effect on PARP-1 or pADPr levels (striped bar; D, F respectively) compared with basal controls (white bar; D, F). Student's t-test (B,D,F); * = p<0.05; ** = p<0.01; NS = not significant.

**Figure 9:**
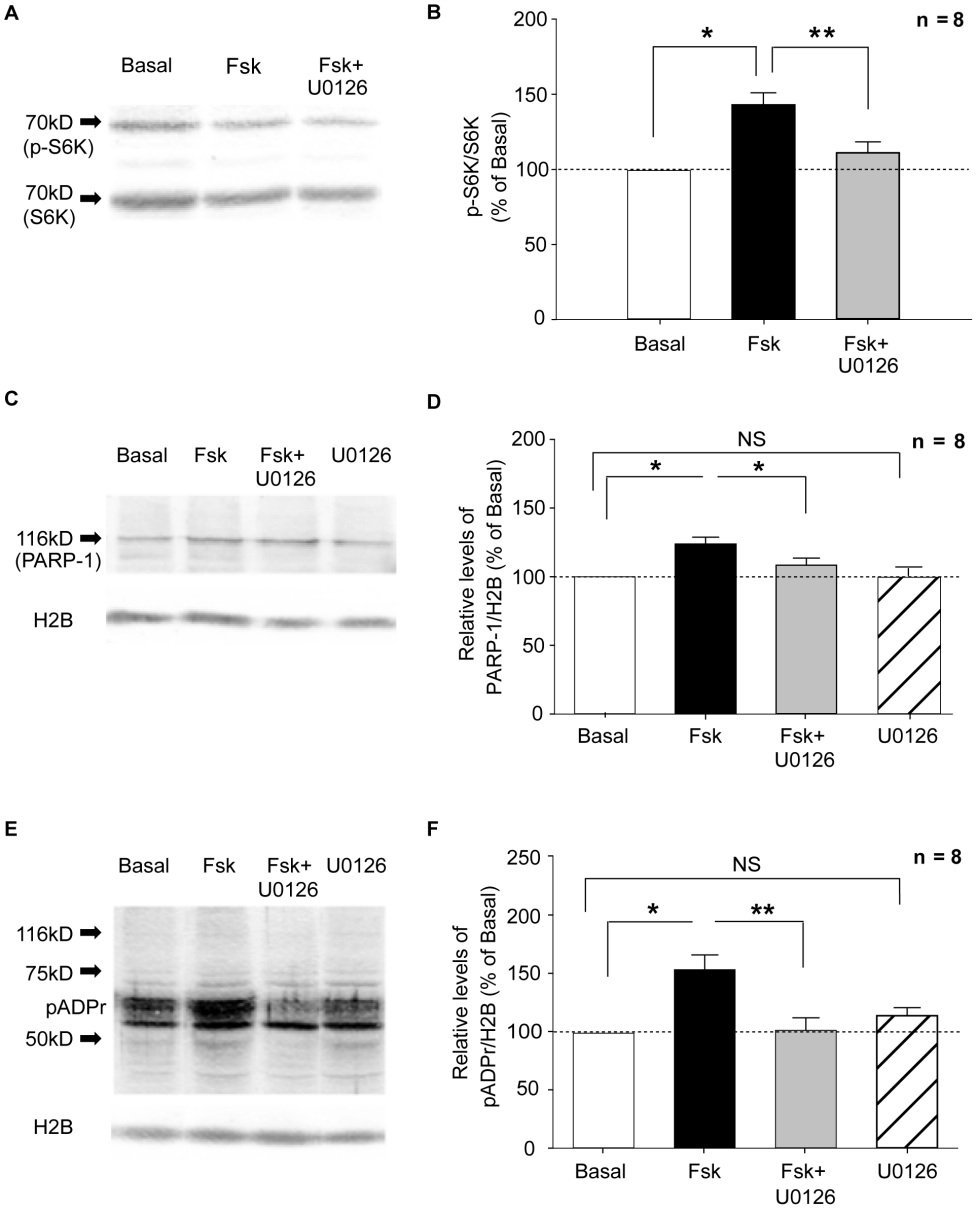
Forskolin-induced increase of PARP-1 and pADPr requires the ERK pathway. Left column (A, C, E): Representative Western blots used for quantification shown in right column (B, D, F). Phosphorylation of the ERK pathway substrate S6K was used as a stimulation-dependent positive control quantified as the ratio of p-S6K to total S6K (A, B). Histone 2B was used as a loading control (C, E). Fsk treatment (black bar) produced an increase of p-S6K, PARP-1, and pADPr (B, D, and F, respectively). The Fsk induced increase was prevented by the ERK inhibitor U0126 (grey bar; B, D, F). The inhibitor alone had no effect on PARP-1 or pADPr levels (striped bar; D, F respectively) compared with basal controls (white bar; D, F). Student's t-test (B,D,F); * = p<0.05; ** = p<0.01; NS = not significant.

3- aminobenzamide (3-AB; Sigma, St. Louis, MO) was used to inhibit PAR (Figs. 1, 2 D,E and 3). Actinomycin-D (Act-D; ICN Biomedicals, Aurora, OH) was used to inhibit RNA polymerase I (Pol I) directed rRNA transcription (Figs. 4, 5, 6, and S2). CX-5461 (Selleckchem, Houston, TX) was used to inhibit Pol I (Fig. 7). Forskolin (Fsk; Calbiochem, La Jolla, CA) was used to evoke a form of long-term potentiation (LTP) (Figs. 2, 3, 6, 8, and 9). Specific kinase inhibitors KT5720 (Sigma) and U0126 (Selleckchem, Houston, TX) were used to block PKA and ERK kinases (Fig. 8 and 9, respectively). All the drugs (stock solutions) were dissolved in dimethyl-sulfoxide (DMSO). Whenever DMSO was used, the final concentration never exceeded 0.05% (v/v).

### Animal preparations

All procedures were approved by the Institutional Animal Care and Use Regulations of SUNY Downstate Medical Center.

#### Cultured neurons (Figs. 1A–G, 4, 7A–I, and S2)

Primary hippocampal neurons were prepared from Sprague-Dawley E18/19 rats (Hilltop), according to the method described by Brewer and collaborators (1993) [16] and were utilized after 14 to 18 days *in vitro*.

#### Hippocampal slice preparation (Figs. 2, 3, 5, 6, 7L–O, 8, and 9)

Adult (2–4 months old) male C57BL6 mice (Taconic Farms) were transferred from the home cage to the anesthetizing chamber and remained in the covered chamber for 15–20 min for acclimation. Subsequently, animals were deeply anesthetized with 5% vaporized isoflurane in oxygen (100%) for 2–3 min and euthanized via decapitation. The brain was removed and rapidly transferred to ice cold, oxygenated (95% O2, 5% CO2) dissection artificial cerebrospinal fluid (dACSF, containing in mM: 125 NaCl, 2.5 KCl, 0.5 CaCl2, 7 MgSO4, 1.25 NaH2PO4, 25 NaHCO3, and 10 Glucose; pH 7.4) and both hippocampi were dissected out. Isolated hippocampi were transversally sliced with a pre-chilled manual tissue chopper (MyNeurolab.com, USA) to produce 400 µm sections. The slices were rapidly transferred to a beaker with oxygenated recording ACSF (rACSF, containing in mM: 125 NaCl, 2.5 KCl, 2 CaCl2, 1 MgSO4, 1.25 NaHPO4, 25 NaHCO3, 25 Glucose) at 35°C for 45 min and then left to recover at room temperature for at least 60 min. After recovery slices were transferred to a recording interface chamber for electrophysiology experiments (to detect LTP expression) or underwent different treatments (see treatments of hippocampal slices for immunohistochemistry, Real-time PCR and Western blot analyses).

### Electrophysiology

Transverse hippocampal slices (400 µm) were incubated in an interface chamber at 34–35°C, subfused with oxygenated rACSF, and allowed to equilibrate for at least 60 min. Field excitatory postsynaptic potentials (fEPSPs) were recorded at CA3/Schaffer collateral-CA1 synapses by placing both stimulating and recording electrodes in the stratum radiatum of the CA1 area. The stimulation intensity (square pulse, 50 µsec duration) was adjusted to give fEPSP slopes of approximately 40% of maximum. Baseline and after-stimuli responses were sampled once per min at this intensity for the duration of the experiment. In order to assess the effect of PAR inhibition on the late stages of LTP (Fig. 2D, E), 3-AB (100 µM) was administered 30 min after LTP–eliciting Fsk treatment (50 µM, 30 min) for 45 min duration. In order to assess the effect of Pol I inhibition on LTP, a low concentration (100 nM) of Act-D (Fig. 6) or (200 nM) CX-5461 (Fig. 7L,M) was added to slices 15 min prior to Fsk treatment and was maintained for the duration of the treatment (45 min total). None of the drugs (3-AB, Act-D nor CX-5461) had an effect on baseline conditions.

### Treatments of hippocampal slices for immunohistochemistry, Real-time PCR and Western blot analysis

#### Treatment to detect whether Fsk induced changes in the expression and distribution of PARP-1 and pADPr (Fig. 2A)

After recovery (see *Hippocampal slice preparation* section), slices were subjected to Fsk (50 µM) or left untreated (rACSF) for 30 min and then collected and prepared for immunohistochemistry (IHC).

#### Treatment to detect whether low-dose Act-D caused nucleolar disruption in mouse hippocampal slices (Fig. 5)

After recovery, slices were treated with Act-D (100 nM) or left untreated (rACSF only) for 45 min and then collected and prepared for IHC.

#### Treatment to detect whether Fsk-induced rRNA and c-jun gene expression (Fig. 3 and 7N,O) required PAR and Pol I activity

Fsk stock solution was diluted in a larger volume of oxygenated rACSF (10 ml) taken from the treatment beaker and then added back for the final 40 ml of 50 µM working solution. The addition of Fsk marked the beginning of treatment (T0). To test whether PAR is required for rRNAs and *c-jun* expression, 3-AB solution (100 µM final concentration) was administered to the appropriate treatment beakers 15 min prior to the addition of Fsk (Fig. 3). The same 15 min pretreatment protocol was performed for the Pol I inhibitor CX-5461 (Fig. 7 N,O). Fsk+inhibitor (3-AB or CX-5461) treated slices were compared with control (no drug) and Fsk treated slices. 3-AB treated slices remained in the presence of drugs until the end of Fsk treatment (T30) at which time they were rapidly transferred from the treatment beakers to beakers containing oxygenated rACSF for a 15 min washout period and then collected. CX-5461 treated slices remained in the presence of drugs until the end of Fsk treatment (T30) at which time they were collected. Control slices remained in the same beakers in the presence of vehicle (DMSO) in oxygenated rACSF for the duration of the experiment. At the end of the treatment, the slices were collected in TRIzol© reagent (Invitrogen) and prepared for RNA isolation and PCR.

#### Treatment to detect whether Fsk-induced PARP-1 and pADPr upregulation required PKA and ERK activity (Fig. 8 and 9)

Mouse hippocampal slices were prepared as described above and drug treatments were conducted as summarized in Fig. S1. After treatment, slices were homogenized and prepared for Western Blot analysis.

### Selection of hippocampal sections for IHC quantification

We analyzed the entire CA1 region (from CA2 to the subiculum) of the hippocampus as indicated in the text and figure legends. We performed 10 experiments. Each experiment utilized a single mouse from which hippocampal slices and microsections were obtained. To avoid variability in the quantification (Figs. 2 and 4) hippocampal slices from the same mouse were incubated with or without drugs (refer to Hippocampal slice preparation and Drug treatment sections above). The comparison control vs treated was always within the same experiment and not between experiments. In addition, the IHC for control and treated slices from the same experiment were done at the same time. Briefly, mouse hippocampal slices (400 µm thick) were collected and fixed overnight in 4% paraformaldehyde (PFA). After fixation, the slices were rinsed with PBS, embedded in a 4% agarose block and microsectioned to 40 µm using a vibratome. The first two 40 µm vibratome sections were discarded to eliminate outer layers containing a high proportion of dead or damaged pyramidal cells. Then, four or five 40 µm thick sections (without vibratome artifacts) were selected for IHC. After IHC, the nucleic acid fluorescent stain DAPI (4′,6-diamidino-2- phenylindole) was observed under the confocal microscope to identify damaged sections. If cell damage occurred during slice preparation, then nuclear DAPI staining would become condensed and the nuclei, pyknotic. Only the sections that had good DAPI staining were quantified.

### IHC for mouse hippocampal slices

#### IHC of PARP-1 and pADPr detection (Figure 2A)

Fsk treated and untreated (control) hippocampal slices were collected, fixed and microsectioned as described above. Microsectioned slices were transferred directly to quenching solution (0.1% Glycine in PBS) for 10 min, rinsed 2x (10 min) with phosphate buffered saline (PBS: distilled water, 0.435% NaH2PO4, 0.973% Na2HPO4, 0.9% NaCl, and 0.01% Na Azide; pH 7.4) and transferred to background suppressor (0.1% Na Borohydride in PBS) for 10 min. The slices were rinsed with PBS 3x (10 min), incubated in blocking solution (2% BSA, 0.1% Triton X-100 with 5% Normal Goat Serum, NGS, in PBS) for 2 h. The slices were then incubated overnight with primary antibody (AB): anti-pADPr polyclonal (1∶200; Cat # 4366-BPC- 100, Trevigen) or anti-PARP-1 polyclonal diluted in blocking solution (1∶200; Cat # sc-7150, Santa Cruz BioTechnology) or incubated in blocker alone (no primary AB control or NP). The slices were then rinsed with PBS 3x (10 min) and incubated overnight in secondary AB (Goat Anti-Rabbit Alexafluor 568; 1∶200) in incubation buffer (0.1 M PBS with 2% BSA and 0.1% Triton X-100) in the presence of DAPI. The next day slices were rinsed in PBS 4x (10 min), rinsed 1x with distilled water (5 min) and mounted onto super frost slides.

#### Fibrillarin detection (Figure 5)

Treated (100 nM Act-D, 45 min) and untreated hippocampal slices were collected, fixed and microsectioned as described above. To expose the fibrillarin epitope, the 40 µm sections were treated with 10 mM sodium citrate buffer (pH 9.0) for 30 min at 80°C [17], washed in PBS (15 min), transferred to quenching solution for 10 min and incubated in PBS with 0.7% Triton X-100 for 15 min. The slices were rinsed with PBS 3x (10 min), incubated in blocking solution (0.1% Triton X-100 with 4% NGS, in PBS) for 2 h and then incubated overnight in anti-fibrillarin polyclonal AB (1∶500; Cat # ab5821, Abcam) diluted in blocking solution or blocker alone (NP). Next, the slices were rinsed with PBS 3x (10 min) and incubated with secondary AB (Goat Anti-Rabbit Alexafluor 488; 1∶200 Molecular probes) for 4 hours before being rinsed again with PBS 3x (10 min), 1x with distilled water (5 min) and mounted with antifade (DAPI Fluoromont-G; Southern Biotech).

### RNA isolation and reverse transcription

#### RNA isolation

Total RNA was isolated from individual hippocampal slices using TRIzol© reagent (Invitrogen), precipitated with isopropanol, resuspended and digested with 1 Unit of DNAse I (Promega) for 30 min at 37°C. The total RNA was re-purified with TRIzol and re- suspended in 10 µl of DEPC-treated water. The RNA purity and concentration was assessed by its 260/280 optical density (OD) ratio.

#### Reverse Transcription

Five microliters of the re-suspended RNA was used for reverse transcription using the Superscript© III First-Strand Synthesis kit protocol for random hexamers (Invitrogen). The cDNA samples were then treated with RNase H for 20 min in order to remove residual RNA. The cDNA concentration was estimated from the initial OD values assuming 80% efficiency of the reverse transcription reaction. Each cDNA sample was diluted to render a 0.3 ng/µl working solution for analysis by quantitative (real-time) PCR (qPCR).

#### qPCR for rRNA and c-jun gene expression

Comparative Ct qPCR was carried out using SYBR-Green RT-PCR Master Mix detection reagent (Applied Biosystems) and Stratagene's Mx3000P Real-Time PCR system. The cDNA was generated using the random hexamer primers provided in Invitrogen's Superscript© III First Strand Synthesis kit (Life Technologies). We used 1.5 ng of cDNA as input for each 20 µl PCR reaction. The final primer concentration was 0.5 µM. In order to quantify stimulation-dependent changes in target gene expression, samples were normalized to the housekeeping gene GAPDH, found to be constitutively abundant in the hippocampus [18]. The primers used for mouse GAPDH were: mGAPDH F 5′-TTGTGATGGGTGTGAACCACGAGA-3′ and mGAPDH R 5′-GAGCCCTTCCACAATGCCAAAGTT-3′. In order to distinguish newly synthesized rRNAs from pre-existing rRNAs we designed primers to a region of heterogeneous pre-rRNA (ht rRNA) between the internal transcribed spacer 2 (ITS2) and the 28S rRNA. The editing of ITS2 from 28S rRNA is one of the rate limiting steps in rRNA maturation. ht rRNA: Acc# X82564 ht rRNA-F 5′-GCCGGGTGCCGTCTCTTT-3′ and ht rRNA-R 5′-TATGCTTAAATTCAGCGGGTCGCC-3′. The immediate early gene, *c-jun* was employed as an activity-dependent positive control. Primer sequences were: mouse *c-jun* F 5′-GAACTGCATAGCCAGAACACGCTT-3′ and *c-jun* R 5′- TGAAGTTGCTGAGGTTGGCGTAGA-3′. The thermocycling conditions were as follows: 50°C for 5 min (1 cycle), 95°C for 10 min (1 cycle), 60°C for 1 min (1 cycle), and 95°C for 15 s followed by 60°C for 1 min (40 cycles). At the end of the protocol, a dissociation curve analysis was performed to determine the specificity of amplification with a start temperature of 55°C.

#### Western blot analysis for PARP-1 and pADPr quantification

After treatment, individual slices were transferred to 70 µL ice-cold homogenization buffer (1% SDS (w/v)), 10 mM EDTA, 50 mM Tris, pH 8.1, and one Roche Protease inhibitor cocktail tablet). An aliquot of the homogenate was taken for protein quantification using a Pierce BCA Protein Assay Kit (Thermo Scientific). The homogenates were immediately diluted (1∶1) in 2x sample buffer (0.125M Tris, 5% SDS (w/v), 10% BME (v/v), 20% glycerol (w/v), 0.01% blue bromophenol (w/v)), denatured and subjected to 10% SDS-polyacrylamide gel electrophoresis. Total protein (15 µg) was loaded into 0.35 cm wells. The proteins were transferred to nitrocellulose membrane. For anti-S6K anti-p70 S6Kinase (S6K) and anti-Phospho-p70 S6Kinase Thr421/Ser424 (p-S6K) the membranes were blocked (1 h) with 5% bovine serum albumin (BSA) in TBS-Tween 20 (TBST) (20 mM Tris-HCl, pH 7.6, 15 mM NaCl and 0.1% Tween 20). For anti-pADPr, anti-PARP-1 and anti-Histone2B (anti-H2B) membranes were blocked (1 h) in 2% BSA and 2% hemoglobin in TBST. After blocking, the membranes were incubated with primary AB overnight at 4°C, washed 3x (5 min) with TBST, incubated with secondary AB in TBST (1 h) and washed 3x (5 min) with TBST. The membranes were developed with BCIP/NBT phosphatase substrate (KPL) until the bands showed clearly. Finally, the membranes were dried, scanned and stored as JPEG files. The band intensity was quantified using ImageJ software (http://rsb.info.nih.gov/ij/). The level of pADPr and PARP-1 was standardized to that of Histone 2B.

Primary ABs: anti- pADPr polyclonal (1∶500, Trevigen); anti-PARP-1 polyclonal H-250 (1∶500; Cat # 7150, Santa Cruz,); anti-p70 S6Kinase (1∶1000; AB Kit Cat # 9430, Cell Signaling); anti-Phospho-p70 S6Kinase Thr421/Ser424 (1∶1000; AB Kit Cat # 9430, Cell Signaling); and anti-H2B (1∶2000; Cat # 07-371, Millipore). Secondary AB: anti-rabbit coupled to alkaline-phosphatase (1∶2000; Cat # A3687, Sigma). BCIP/NBT phosphatase substrate (KPL) was used as the detection reagent.

### Immunocytochemistry (ICC) for primary hippocampal cultures

#### Fibrillarin ICC

Cultured neurons were treated with or without 3-AB (400 µM final, 3 h; *Fig. 1A–G*) or CX-5461 (200 nM final, 1 h and 3 h; *Fig. 7A–I*) and then fixed for 10 min in 4% PFA in PBS (pH 7.4). The cultures were washed with PBS 3x (10 min) and incubated in 1% SDS in PBS (10 min) to improve nuclear immuno-detection [19]. The cultures were then washed with PBS 4x (15 min), incubated in blocking solution (0.1% Triton X-100, 2% NGS in PBS) for 1 h and then incubated overnight with primary AB (polyclonal anti-fibrillarin AB (1∶1000); Cat # ab5821, Abcam) in blocking solution or blocking alone (NP control). The cultures were rinsed with PBS 4x (10 min) and then incubated with secondary AB (GAR 568) for 4 h in darkness. After secondary AB incubation, the cultures were rinsed with PBS 4x (10 min), rinsed 2x with distilled water (5 min each), and mounted with antifade (DAPI Fluoromont-G; Cat # 0100-20; Southern Biotech).

#### Double ICC for fibrillarin and PARP-1 (Fig. 4)

Cultured neurons were treated with or without low concentrations of Act-D (100 nM final) for 60 min and then fixed for 10 min in 4% PFA in PBS (pH 7.4). The cultures were washed with PBS 3x (10 min) and incubated in 1% SDS in PBS (10 min) to improve nuclear immuno-detection [19]. The cultures were then washed with PBS 4x (15 min), incubated in blocking solution (0.1% Triton X-100, 2% NGS in PBS) for 1 h. With the exception of the NP control, all cells were incubated overnight with primary ABs (mouse anti-PARP-1 (1∶500); Cat # MCA1522G, AbD Serotec and polyclonal anti-fibrillarin AB (1∶1000); Cat # ab5821, Abcam) in blocking solution. The NP cells were incubated in blocking solution (4% NGS/PBS, 0.1% Triton X-100) without AB overnight. The cultures were rinsed with PBS 4x (10 min) and then incubated with secondary AB (GAR 488 and GAM Biotin 1∶200 dilution) for 4 h at room temperature in darkness. After secondary incubation, the cultures were rinsed with PBS 4x (10 min) and incubated with Strep Alexa 647 (far red) for 2 h in darkness at room temperature, rinsed with PBS 4x (10 min), rinsed 2x with distilled water (5 min) and mounted with antifade (DAPI Fluoromont-G; Cat # 0100-20; Southern Biotech).

#### Double ICC for fibrillarin and nucleostemin for supplementary figure 2 (Fig. S2)

Cultured neurons were treated with or without low concentrations of Act-D (100 nM) for 3 h at 37°C to specifically block RNA polymerase I. After treatment, the cultures were rinsed with warm 37°C PBS and fixed for 10 min in 4% PFA in PBS, pH 7.4. Following a 10 min wash in PBS, cells were permeated with 1% SDS in PBS solution for 10 min. After 4x (15 min) washing cycles with PBS, cells were incubated in blocking solution for 1 h. The blocking solution was retrieved and cells were washed with PBS 2x (10 min) following a 1 h of incubation. With the exception of the NP control, all cells were incubated overnight with the first primary AB, rabbit anti-antigen [Rp pAb to fibrillarin; Abcam5821 (1∶1000)] in blocking solution. The NP cells were incubated in blocking solution overnight. The primary AB and NP were retrieved following overnight incubation and cells were washed with PBS 4x (10 min). Cells were then incubated overnight with secondary AB, GAR 568, for 4 h in darkness. Cultures were then washed with PBS 4x (10 min) and incubated in normal rabbit serum for 1 h. Next, cultures were washed with PBS 3x (10 min) and incubated with Fab goat anti-rabbit IgG (H+L) (1∶160) in 1X PBS, 0.1% Triton X-100 for 1 h. Cultures were then washed with PBS 4x (10 min) and incubated overnight with the second primary AB Rp pAB to nucleostemin, Cat # 70346 Abcam (1∶1000)] diluted in blocking solution (4% NGS/PBS, 0.1% Triton X-100) overnight on rocker. Following overnight incubation, cultures were washed with PBS 4x (10 min) and incubated overnight in darkness with GAR (488). On the next day, cultures were rinsed with PBS 4x (10 min), then with sterile water 2x (5 min). Cultures were then mounted on slides with antifade (DAPI Fluoromont-G; Cat # 0100-20; Southern Biotech) and cured overnight on a flat surface. Slides were then stored at −20°C until imaging analysis.

### Image analysis for fibrillarin, PARP-1, and pADPr

Images were analyzed using Olympus Fluoview ver. 2.1a (FV10-ASW version 02.01.01.04). All regions of interest (ROIs) were drawn and analyzed blind to control and treated conditions. All images in a given experiment were shot using identical optical and digital magnification, laser intensities, photomultiplier voltages, and amplifier gains which were selected to give optimal dynamic range for the experimental conditions without saturation in any channel. For quantification of average intensity of nucleolar PARP-1 (see representative Fig. 4) ROIs were determined by the extent of fibrillarin staining. For Fig. 5C, F and Fig. 1I, J, the area and average fibrillarin intensity in were quantified using ROIs determined by the extent of fibrillarin staining. For Fig. 7J, K only the largest continuous area of fibrillarin staining in each nucleus was used as an ROI as the fibrillarin would break up into many smaller punctate regions at later times. For pADPr and PARP-1 (Fig. 2A–C), ROIs were determined from the extent of DAPI staining and the average intensities of PARP-1 (Fig. 2B) and pADPr (Fig. 2C) were quantified. Intensity measurements were taken on a 0-4095 pixel intensity scale and results were averaged and controls normalized to one. Representative images were chosen for figures.

#### Statistics

For all statistical tests a difference between tested groups was considered significant if the p<0.05. For immunohistochemistry and immunocytochemistry, homoscedastic two tailed t-tests were performed. For Western Blot and qPCR analysis, two-tailed paired Student's t-tests were performed. For electrophysiology, two-way repeated measure ANOVA and pairwise multiple comparison procedure Student-Newman-Keuls analyses were performed using SigmaStat statistical software (Systat Software. Richmond, CA, USA). Data in figures was presented as mean ± SE.

## Results

Ribosomal RNA is expressed and processed in the nucleolus, a subnuclear structure functionally organized around rDNA transcription and ribosomal biogenesis [20]. The morphology of the nucleolus is inextricably linked to its function. The nucleolus is organized into three distinct functional compartments—each corresponding to a specific stage in the synthesis and processing of rRNAs and their assembly into ribosomes [21], [22]. Accordingly, changes in nucleolar integrity reflect changes in nucleolar function. PARP-1 is highly abundant in nucleoli and is now known to play pivotal roles in nucleolar activities such as rRNA processing and ribosome biogenesis [15]. PARP-1 has been found to be crucial for the maintenance and transmission of rDNA chromatin structure and for the proper organization of heterochromatin during development [14] [23]. Moreover, studies in *Drosophila* have demonstrated that in the absence of *Drosophila* PARP (dPARP) gene expression, nucleoli themselves fail to form. This disruption can be rescued, and nucleolar formation restored, with the ectopic expression of dPARP [24].

In a previous study we showed that PARP-1 activity was required for the maintenance of long-term synaptic plasticity in *Aplysia*, as well as the plasticity evoked upregulation of rRNA [11]. Given the evidence for the importance of PAR to both nucleolar function and learning and memory, we hypothesized that epigenetic regulation of rDNA expression by PARP-1 is necessary for long-term synaptic plasticity and memory in mammals. To begin the testing of our hypothesis we proceeded to investigate the relationship of PARP-1 and nucleolar function to long-term synaptic plasticity in mice.

### Inhibition of PAR results in increased intensity of the nucleolar protein, fibrillarin

We began by asking, what is the effect of PAR inhibition on the functional integrity of neuronal nucleoli? A hallmark of Pol I inhibition and nucleoli disruption is the fragmentation of nucleolar compartments which can be tracked through the localization of nucleolar proteins such as fibrillarin or nucleostamin. It has been shown that the nucleolar retention of fibrillarin, a protein involved in the processing of precursor rRNAs, depends on active rRNA synthesis by Pol I [25]. In order to examine the relationship of PAR to functional nucleolar compartments, we applied the PAR inhibitor, 3-Aminobenzamide (3-AB; 100 µM, 3 h), to dissociated hippocampal neuron culture (Figure 1). Since dPARP disruption affects nucleoli formation and ectopic expression of dPARP can restore nucleolar formation in drosophila [24], we expected, in our studies, that PAR inhibition would affect nucleoli integrity. Measuring the average area of fibrillarin as a marker of nucleolar integrity, we found no significant difference between 3-AB treated and untreated control cultures (Fig. 1, compare A, C to B, D); suggesting that PAR inhibition does not substantially affect nucleolar integrity (Fig. 1I). However, in the same cells we did find significant changes in the intensity of fibrillarin staining with 3-AB treated cells (Fig. 1, compare G to H) showing about a 60% increase in average intensity compared to untreated control (Fig. 1J, Student's t-test p = 0.0004); suggesting an altered state of the nucleoli in the presence of 3-AB.

### PAR is required for the expression of long-term synaptic plasticity in mouse hippocampus

PAR inhibition did not produce the substantial and well-defined structural changes that characterize nucleolar disruption, however, PARP-1 has been shown to be required for LTF in *Aplysia* [11]. Therefore, we tested the functional relevance of PAR during LTP. We examined whether: 1) PARP-1 expression and activity (PAR) is regulated by mechanisms of synaptic activity in the mouse hippocampus; and 2) PAR is required for the maintenance of LTP. To address the first question, we used immunohistochemistry (IHC) to determine whether forskolin (Fsk) treatment that induces LTP in mouse hippocampal slices alters expression and localization of PARP-1 and pADPr (Fig. 2A). We found that Fsk (50 µM) treatment caused a dramatic increase in the intensity of the nuclear localization of both PARP-1 (Fig. 2B, Student's t-test, p = 7.41E-20) and pADPr (Fig. 2C, Student's t-test, p = 3.704E-5) in CA1 pyramidal cells of mouse hippocampal slices, supporting the assertion that PARP-1 is regulated by mechanisms linked to activation of synaptic activation in the mouse hippocampus. In order to specifically assess the role of PAR in later stages of LTP, we applied the PAR inhibitor, 3-AB (100 µM, 45 min), 30 min after Fsk treatment (Fig. 2D,E). We found that late expression (>3 h) of Fsk-induced LTP is significantly blocked by 3-AB (Fig. 2E, Student-Newman-Keuls t-test, p = 0.004). These data indicate a functional requirement of PAR for the persistence of Fsk-induced LTP.

### PAR is required for plasticity-dependent expression of rRNAs

Next we asked, what is the relationship between stimulation-dependent PAR and nucleolar function as indicated by rRNA gene expression? To address this, we measured the expression of nascent precursor rRNA by quantitative (real-time) PCR in hippocampal slices treated with Fsk (50 µM, 30 min) in the presence and absence of 3-AB (100 µM) compared to untreated control slices (Fig. 3). To differentiate between Fsk-induced rRNAs and the pre-existing rRNA pool, we designed primers specific for the heterogeneous (unedited) pre-rRNA transcript (ht rRNA). As a positive activity-dependent control, we measured the expression of the immediate early gene (IEG) *c-jun*, whose inducible expression has been shown to be regulated by PARP-1 [13]. We found an 80% increase in ht rRNA levels in Fsk-only treated slices compared to controls (Fig. 3A, Student's t-test p = 0.002). In the presence of 3-AB, the Fsk-induced increase of precursor rRNA was significantly reduced (Fig. 3A, Student's t-test p = 0.005). As expected, *c-jun* expression was significantly increased in response to Fsk treatment (Fig. 3B, Student's t-test p = 0.03)—an upregulation that was suppressed in the presence of 3-AB (Fig. 3B, Student's t-test p = 0.02). These results suggest that the nucleolus participates in the neuronal response to potentiating stimulation and that plasticity dependent rRNA expression is regulated by PAR.

### Low concentration Actinomycin D treatment disrupts the nucleolar localization of PARP-1

So far we have shown that PAR is required for the late expression of LTP (Fig. 3D) and for the stimulation-evoked upregulation of rRNA (Pol I dependent transcription) (Fig. 3A). However, it has already been established that inhibition of PAR also affects IEGs (Pol II dependent expression) during Fsk-induced LTP (Fig. 3B) and long-term memory formation [13], leaving it difficult to ascertain whether activity dependent Pol I transcription is necessary for LTP. In order to focus on nucleolar function (rRNA synthesis), we employed low concentration of the transcriptional inhibitor, Actinomycin D (Act-D) (Fig. 4). Numerous studies have demonstrated that low concentrations (8 nM to 800 nM or 0.01 µg ml^−1^ to 1 µg ml^−1^) of Act-D selectively blocks Pol I directed rDNA transcription while leaving Pol II and Pol III mediated transcription intact [25]–[31]; reviewed by [32] and [33]. Employing low dose Act-D (100 nM) treatment in dissociated hippocampal neuron cultures, we found that a 1 h exposure was sufficient to cause fibrillarin to concentrate into small punctate domains indicative of nucleolar disruption (Fig. 4B,D, small arrowheads). Similar results were obtained using nucleostemin as a marker of nucleolar integrity (Fig. S2). Low Act-D treatment dramatically altered the nuclear localization of PARP-1, causing the protein to vacate nucleoli (compare Fig. 4E, large arrowheads with 4F, small arrowheads). There was a 32% decrease in the average intensity of nucleolar PARP-1 staining in Act-D treated cultures compared with untreated controls (n = 10; Student's t-test, p = 7.9E-07). Our observations in primary cultured neurons concur with a study performed in cell lines where Act-D treatment caused PARP-1 to leave nucleoli and become dispersed throughout the nucleus [34]. The dramatic redistribution of PARP-1 out of the nucleolus and into the nucleoplasm suggests that, under these conditions, the nucleolar functions of PARP-1 would be compromised. Taken together, these findings suggest a structure–function link through which PARP-1 regulates the rRNA biosynthesis pathway by participating in the functional organization of nucleolar compartments.

### Low dose Actinomycin D treatment disrupts nucleolar integrity in mouse hippocampal slices

Having shown acute nucleolar disruption with low dose Act-D in dissociated hippocampal neuron culture, we next tested the efficacy of the treatment in pyramidal cells of the CA1 region of mouse hippocampal slices. As shown in Fig. 5, a 45 min treatment of Act-D (100 nM) is sufficient to cause fibrillarin to concentrate into compact punctate domains (small arrowheads Fig. 5B,E) compared to untreated slices (large arrowheads Fig. 5A,D). This resulted in a significant decrease in the average area of fibrillarin staining (Fig. 5C, Student's t-test p = 3.69E-09). Furthermore, the decrease in area coincided with a significant increase in fibrillarin intensity (Fig. 5F, Student's t-test p = 0.001). While we cannot exclude the possibility of changes in fibrillarin expression, the increase in intensity coupled with a reciprocal decrease in area suggests a change in the distribution of fibrillarin within nucleolar compartments indicating nucleolar disruption.

### Nucleolar integrity is required for late expression of LTP in mouse hippocampal slices

To determine whether nucleolar integrity is necessary for long-term synaptic plasticity we used the same treatment protocol as in the previous experiment (Fig. 5) to temporarily disrupt functional nucleolar compartments during Fsk-induced LTP (Fig. 6). Application of Act-D (100 nM, 45) 15 min before and during Fsk treatment blocked the persistence of LTP (Fig. 6A). Note that although Act-D is present during LTP induction, only its late expression (2 h) was impaired (Fig. 6B, Student-Newman-Keuls test p = 0.007). These results suggest that nucleolar integrity is required for long-term synaptic plasticity.

### The specific Pol I inhibitor, CX-5461, causes nucleolar disruption, blocks the maintenance of LTP and reduces plasticity-dependent expression of rRNA

Act-D disrupts Pol I activity by targeting GC-rich DNA sequences especially enriched in rDNA promoters [32]. To further restrict our disruption to nucleolar integrity maintained by Pol I activity, we employed a second inhibitor, CX-5461, the first selective inhibitor of Pol I known to directly target the Pol I multienzyme complex [35]–[37]. Like Act-D, CX-5461 has also been shown to cause rapid nucleolar disruption in cell lines [38]. Using cultured hippocampal neurons we found that a 3 h application of 200 nM CX5461 was sufficient to cause fibrillarin to concentrate into small punctate domains indicative of nucleolar fragmentation (Fig. 7C,F). A significant decrease in the average area of fibrillarin staining was seen as early as 1 h (Fig. 7J, Student's t-test p = 0.0002); however, the decrease in area became particularly marked after 3 h incubation (7J, Student's t-test, p = 5.34×10^−8^). We also find significant differences in the average area of fibrillarin staining between 1 h and 3 h groups (7J, Student's t-test, p = 0.0183). No significant changes in fibrillarin intensity were observed (Fig. 7K).

We next examined the functional consequences of CX-5461 on LTP (Fig. 7 L,M) and plasticity dependent gene expression (Fig. 7 N,O). As we did for the Act-D treatment, CX-5461 (200 nM) was added 15 min prior to LTP induction and the inhibitor was maintained for the full 30 min Fsk treatment. The application of CX-5461 had no effect on the baseline recording of treated slices (7M, upper left inset); nor did it subtract from the robust response of slices to forskolin at the onset and early stages of LTP (7L a,b). Only the very late expression (>4 h) of LTP was affected by the application of CX-5461 (Fig. 7M, Student-Newman-Keuls test, (4 h) p = 0.034; (5 h) p = 0.03). To confirm the selectivity and efficacy of *in vitro* CX-5461 treatment, we analyzed the Fsk- inducible expression of Pol II dependent *c-jun* and Pol I dependent rRNA (ht rRNA). As expected, Fsk treatment produced a significant increase in *c-jun* expression immediately following the 45 min treatment (Fig. 7N, Student's t-test, p = 0.007). The Pol I inhibitor CX-5461 had no effect on the Fsk-induced expression of *c-jun* mRNA (Fig. 7N; compare black bar with grey bar). In contrast, the Fsk-evoked increase in rRNA precursor was significantly reduced (7O; compare black bar to grey bar, Student's t-test p = 0.017). These results indicate that nucleolar integrity—and indeed, rRNA biosynthesis, are required for long-term synaptic plasticity.

### PARP-1 and pADPr upregulation is dependent upon PKA activity

Since PAR is required for both the maintenance of Fsk-evoked LTP and the activity-induced expression of rRNAs, we next asked: what molecular pathway leads to the upregulation of PARP-1 and pADPr during LTP? Forskolin is a potent activator of adenylate cyclase which results in the rapid release of cAMP [39]. Cyclic AMP-dependent protein kinase A (PKA) together with MAP Kinase/ERK has been shown to play an essential role in long-term plasticity and memory consolidation in invertebrates and mammals [40]–[43]. Therefore it seemed likely that Fsk treatment would activate PARP-1 through the same phosphorylation cascades. To test this hypothesis, we performed a Western blot analysis of Fsk-treated (50 µM, 30 min) and untreated mouse hippocampal slices (see Fig. S1). Previously, it was shown that the ratio of phosphorylated-p70S6 Kinase (p-S6K) to total p70S6K (S6K) protein increases as a result of Fsk-induced LTP [44]. Therefore, we used the p-S6K/S6K ratio as a positive indicator of Fsk-induced potentiation (Fig. 8A, B). Consistent with the IHC in slices (Fig. 2), we found that both PARP-1 protein (Fig. 8C, D PARP-1, Student's t-test p = 0.047) and pADPr (Fig. 8E, F pADPr, Student's t-test p = 0.029) were significantly elevated in Fsk treated slices compared to controls. The activity-dependent increase in both PARP-1 and pADPr was blocked by the addition of the PKA inhibitor, KT5720 (Fig. 8D PARP-, Student's t-test p = 0.038; 8F pADPr, Student's t-test p = 0.003). These results indicate that the Fsk–induced upregulation of PARP-1 and pADPr requires PKA activity.

### PARP-1 and pADPr upregulation is dependent upon ERK activity

To test for the involvement of ERK, we used a similar protocol to that used for PKA (Fig. S1). As in the previous experiments, we observed a significant increase in PARP-1 and pADPr following Fsk treatment (Fig. 9C, D PARP-1, Student's t-test p = 0.015; 9E, F pADPr, Student's t-test p = 0.029). These increases were effectively blocked in the presence of the ERK inhibitor, U0126 (Fig. 9D PARP-1, Student's t-test p = 0.022; 9F pADPr, Student's t-test p = 0.002). These results indicate that the Fsk–induced upregulation of PARP-1 and pADPr requires ERK activity.

## Discussion

In 1950, Katz and Halstead first proposed that memory formation required new protein synthesis [1]—a hypothesis that was not confirmed in mammals, invertebrates, and *in vitro* models of memory until decades later [2], [45], [46]. In order for memories to become consolidated, new transcription must accompany the new, activity-dependent translation [5], [9]. So far, most efforts to understand experience-induced changes in neuronal gene expression have focused on the transcriptional products of RNA polymerase II (precursor mRNA, snRNA and microRNA). Here we show for the first time that the consolidation and persistence of stimulation-evoked plasticity requires nucleolar integrity and function. In addition, we show that plasticity-dependent upregulation of rRNA synthesis requires PAR.

Developmental biologists have long appreciated the importance of rRNA metabolism as a driver of cell growth and responsivity to environmental cues [47]. Transcription of rDNA, rRNA processing, and ribosome assembly all occur in the nucleolus, a subnuclear structure exquisitely attuned to dynamic growth demands and cellular stressors; reviewed by [47]–[49]. Components of the nucleolus have been found to affect cell regulation in a variety of ways. For example, the organization of rDNA in the nucleolus has been shown to affect global chromatin structure and gene expression throughout the nucleus [50]. PARP-1 has been found to be essential for the inheritance of rDNA chromatin structure [14], the processing of precursor rRNAs and their assembly into ribosomes [15].

### Nucleolar function in neuronal plasticity

As observed in cell growth and development, increased nucleolar activity has been seen to coincide with activity-dependent plastic changes in a number of learning and memory models ranging from spatial learning in goldfish [51] to dissociated primary hippocampal cell culture; reviewed by [52]. Recently it was shown that neurite outgrowth induced by the neurotrophic factor, BDNF, is mediated by Pol I driven transcription resulting in the increased production of ribosomal subunits [53]. In an earlier study, Jordan and colleagues [54] found that sustained neuronal activity results in both an increase in nucleoli and their functional capacity. What was unknown, prior to this study, is that stimulation-dependent rRNA synthesis is required for the maintenance of LTP.

### Regulation of rRNA synthesis during long-term synaptic plasticity

Long-term synaptic plasticity requires a mechanism that can integrate diverse intracellular signaling cascades to coordinate *de novo* transcription and local protein synthesis. In recent years, epigenetic mechanisms such as acetylation, methylation and PAR have been found to be crucial for cognitive processes such as learning and memory; reviewed by Day and Sweatt [55], [56]. PARP-1 becomes activated after training in certain memory tasks and is responsible for the opening of chromatin required for plasticity-dependent gene expression in invertebrates and mammals [10], [12], [13]. Among the plasticity-dependent genes already known to be regulated by PARP-1 are the immediate early genes such as *c-fos* and *c-jun* [13]. Using *c-jun* as a positive control for PAR-dependent gene expression, we demonstrate here that rRNAs are also regulated by PAR during late-phase LTP. Additionally, we found that the expression and localization of PARP-1 and PAR, is regulated by a mechanism of synaptic plasticity—Fsk-induced LTP—via PKA and ERK kinase cascades.

To assess the role of nucleoli in plasticity we disrupted nucleolar function in three ways: 1) 3-AB treatment which blocked regulated plasticity-dependent gene expression; 2) low-dose Act-D treatment which preferentially blocks transcriptional activity at GC–rich rDNA promoters; and 3) application of the highly specific Pol I inhibitor, CX-5461, which directly targets the Pol I multi-enzyme complex [35], [37], [38]. Each drug treatment was expected to directly and/or indirectly target ribosomal biogenesis through its rate limiting step, the production and maturation of rRNA. Each treatment prevented the maintenance of late-phase LTP.

While ribosomal biogenesis remains the primary function around which nucleoli take shape, a role for non-ribosomal functions in plasticity and memory, in addition to the requirement of new ribosomes, cannot be ruled out. For example, it is known that a large number of proteins that have no identifiable link to ribosomal biogenesis accumulate in the nucleolus. It has been postulated that nucleolar compartments might serve as reservoirs through which the nuclear concentration of transcription factors and other “molecular switches,” can be regulated [57]; reviewed by Hetman and Pietrzak [33].

### The importance of nucleolar integrity to plasticity and cognition

Over the past several years, there has been increasing evidence pointing toward nucleolar impairment as a common denominator in diseases effecting memory and cognition such as Huntington's, Parkinsons' and Alzheimers' disease (AD); reviewed by Parlato and Kreiner, [58]. In an important study by Pietrzak et al., [59], it was found that the rDNA promoter was hypermethylated in cerebro-cortical samples from patients diagnosed with Mild Cognitive Impairment (MCI) and AD compared to controls, suggesting that epigenetic silencing of rDNA might play a role in the memory deficits common to the two disorders. Recently, in a study complementary to ours, it was elegantly demonstrated that long-term deficits in nucleolar function indeed compromises learning and memory and that nucleolar integrity is not only neuroprotective, but required for neuronal homeostasis [60]. Our finding that acute nucleolar disruption prevents the maintenance of activity-induced LTP represents a new insight into the molecular mechanism of long-term plasticity and memory consolidation.

### Model for late-phase synaptic plasticity

**Figure 10:**
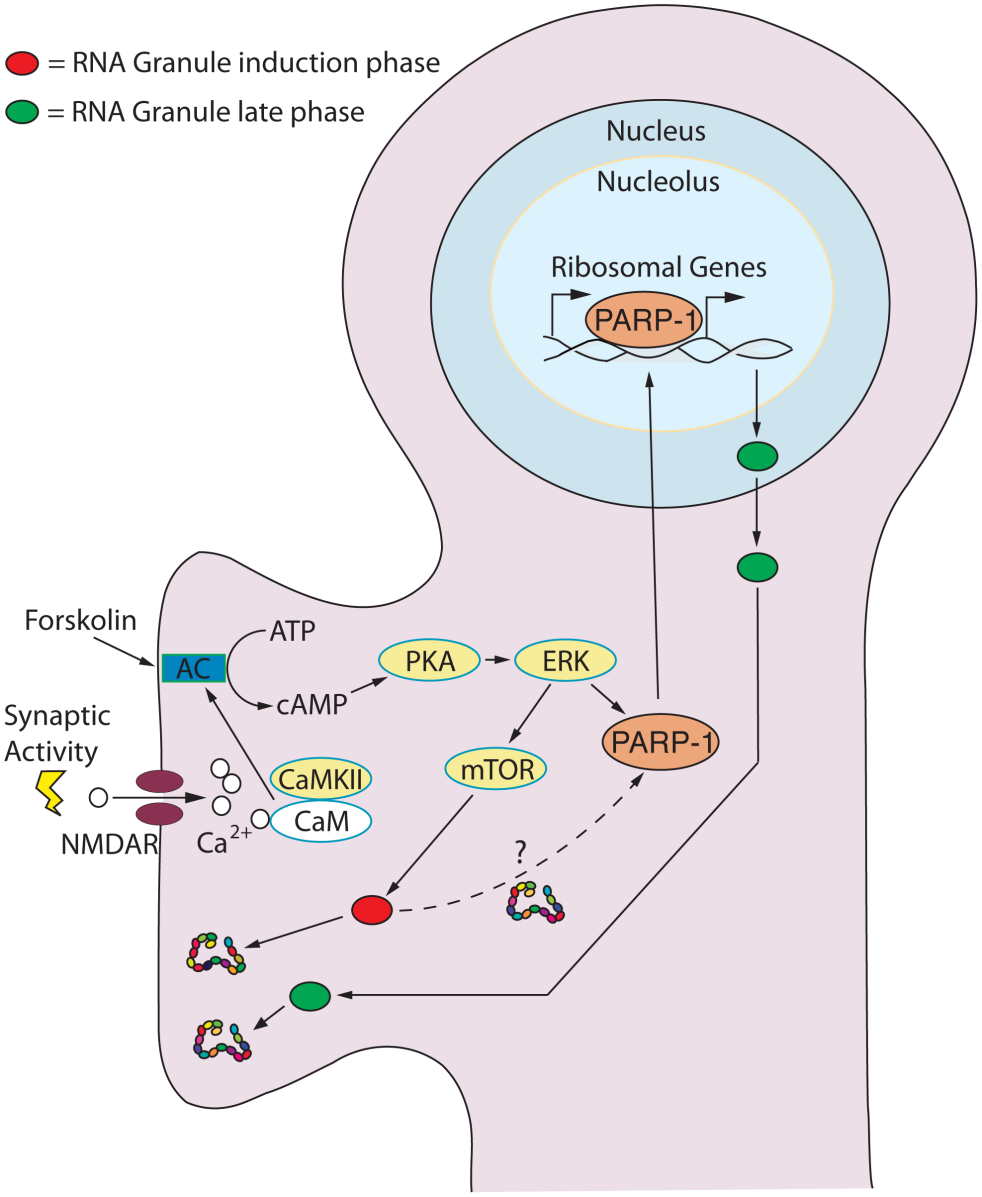
Model for the Role of the Nucleolus in Synaptic Activation leading to Long-Term Plasticity. Synaptic stimulation activates adenylate cyclase (AC) resulting in the rapid release of cAMP and the activation of the cAMP→PKA→ERK pathway (kinases are indicated in yellow). Stimuli leading to long-term plasticity activate mTOR dependent protein synthesis of preexisting RNA granules (red ovals) allowing the transition into late-phase synaptic plasticity. Simultaneously, the cAMP→PKA→ERK pathway induces the synthesis and activation of chromatin remodeling proteins (e.g. PARP-1) and new gene expression. It is not yet known whether the mTOR pathway directly contributes to the activation and up-regulation of PARP-1 (dashed line). PARP-1 opens the chromatin allowing activity-dependent transcription to take place. Among the new genes expressed are activity-dependent Pol I transcripts required for the consolidation of late-phase synaptic plasticity. In this model, the new stimulation-evoked rRNAs give rise to new ribosomes. The new plasticity-dependent ribosomes are assembled into new RNA granules (green ovals) and shipped to activated synapses to maintain, through local protein synthesis, the long-lasting changes required for long-term synaptic plasticity and memory.

Here we present a model (Fig. 10) for the transduction of synaptic stimuli to long-term plasticity beginning with the activation of the cAMP→PKA→ERK pathway. This process is achieved with the activation of mTOR dependent translation of preexisting RNA granules (red) allowing for the transition from short-term plasticity to the initial phase of long-term plasticity. Simultaneously, the cAMP→PKA→ERK pathway induces the synthesis and activation of chromatin remodeling proteins including PARP-1. PARP-1 allows chromatin relaxation and the transcription of immediate early genes (e.g. *c-fos* and *c-jun*) and late-phase genes require for long-term plasticity. An essential component of late-phase activity-dependent gene expression is rRNA biosynthesis, the rate-limiting step in the production of new ribosomes. Ribosomal biogenesis requires both Pol I driven transcription and the efficient processing of nascent rRNA transcripts—two processes now shown to be regulated by PAR. In this model, new and qualitatively different ribosomes become assembled into new RNA granules (green) that are shipped to activated synapses in order to maintain, through local protein synthesis, the long-lasting changes required to consolidate memory.

## Supporting Information

Figure S1 **Drug treatment protocol for PKA and ERK experiments.** For the basal condition control (line A), the slices were kept in oxygenated ACSF for the duration of the experiment. The addition of Fsk marked Time 0 (T0) and lasted for 30 min (line B). For co-treatment with Fsk and inhibitor (KT5720 or U0126), the inhibitor was added 20 min prior to T0 (line C). As a negative control, PKA or ERK inhibitors (KT5720 or U0126, respectively) were applied alone for the duration of the experiment (line D).
(TIF)

Figure S2 **Actinomycin D dramatically disrupts the nucleolar localization of nucleostemin and fibrillarin in cultured hippocampal neurons.** (A,B) DAPI (blue), fibrillarin (red), nucleostemin (green). DAPI staining shows the area of the nucleus. (G, H) Nomarski images (grey) show the area of the nuclei and nucleoli (arrowheads). Low-dose Act-D treatment (100 nM, 3 h) causes nucleolar disruption as indicated by the distribution of fibrillarin and nucleostemin (small arrowheads); compare A,C,E to B,D,F respectively. Bar = 10 µm.
(TIF)
