# Supplementary figures and images for: Nucleolar Integrity Is Required for the Maintenance of Long-Term Synaptic Plasticity
Source: PLoS One. 2014 Aug 4;9(8):e104364. doi: 10.1371/journal.pone.0104364 (PMC4121280; doi:10.1371/journal.pone.0104364)

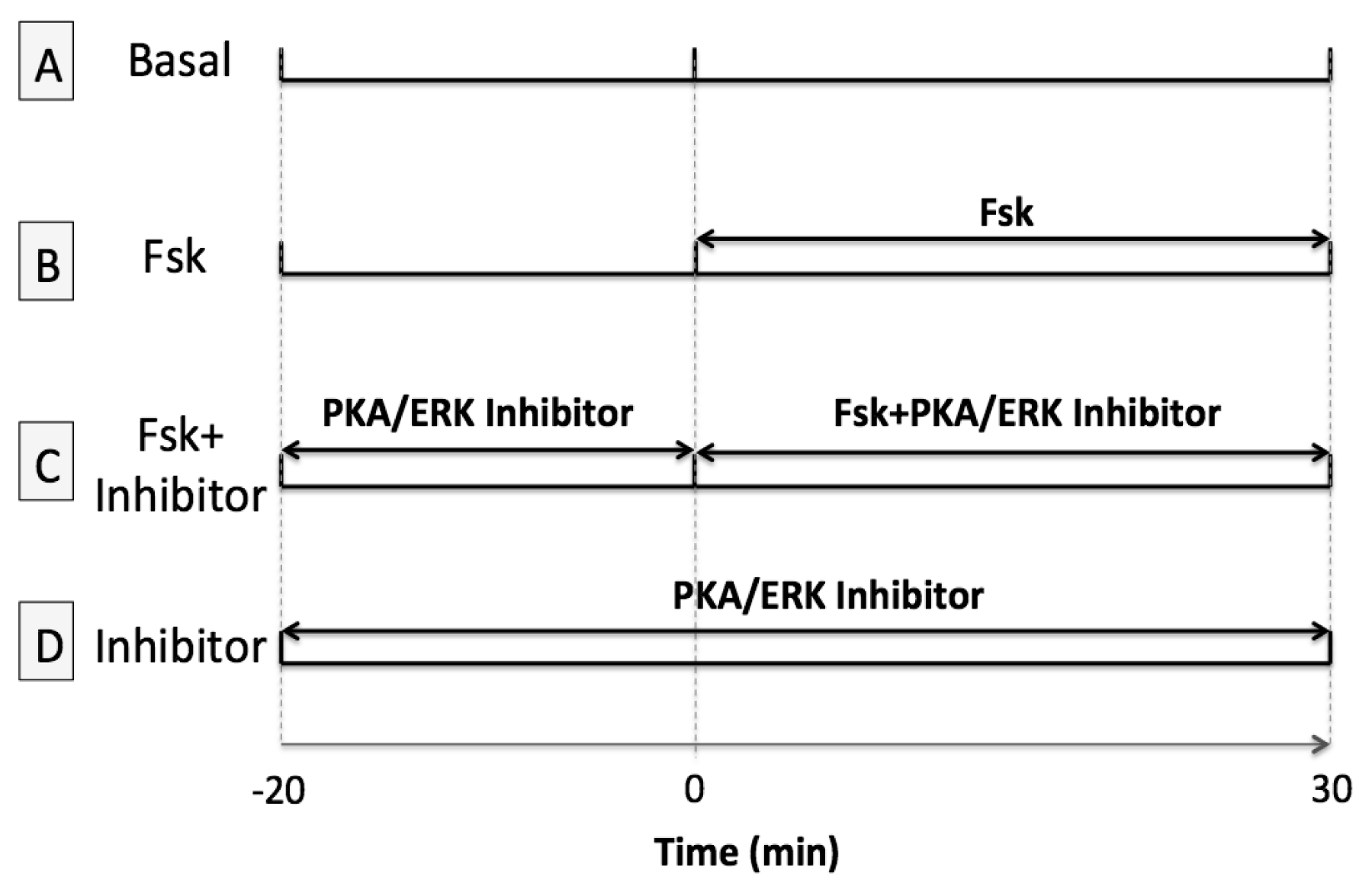

Supplement: Figure S1 — Drug treatment protocol for PKA and ERK experiments. For the basal condition control (line A), the slices were kept in oxygenated ACSF for the duration of the experiment. The addition of Fsk marked Time 0 (T0) and lasted for 30 min (line B). For co-treatment with Fsk and inhibitor (KT5720 or U0126), the inhibitor was added 20 min prior to T0 (line C). As a negative control, PKA or ERK inhibitors (KT5720 or U0126, respectively) were applied alone for the duration of the experiment (line D). (TIF) [file pone.0104364.s001.tif]

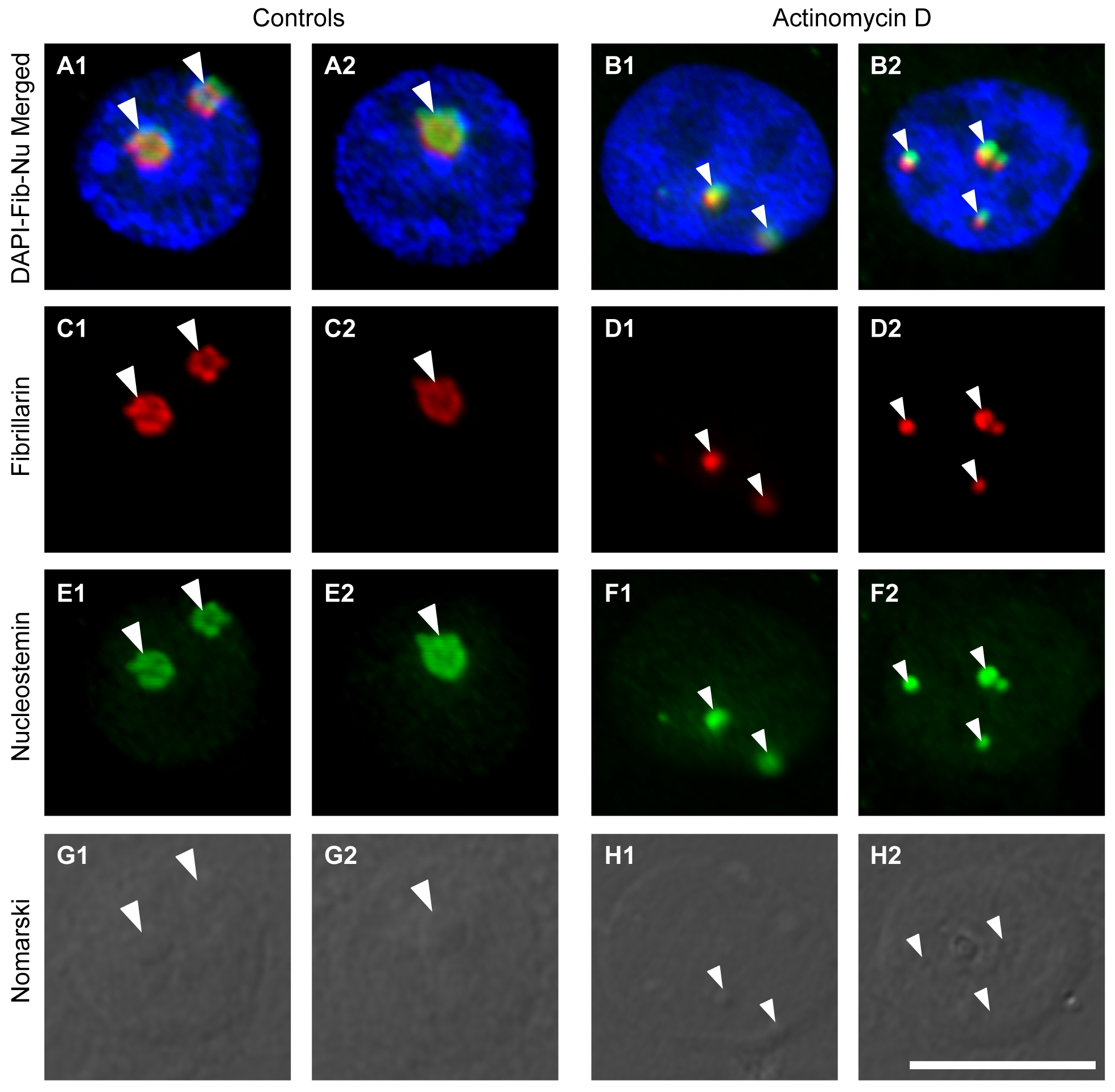

Supplement: Figure S2 — Actinomycin D dramatically disrupts the nucleolar localization of nucleostemin and fibrillarin in cultured hippocampal neurons. (A,B) DAPI (blue), fibrillarin (red), nucleostemin (green). DAPI staining shows the area of the nucleus. (G, H) Nomarski images (grey) show the area of the nuclei and nucleoli (arrowheads). Low-dose Act-D treatment (100 nM, 3 h) causes nucleolar disruption as indicated by the distribution of fibrillarin and nucleostemin (small arrowheads); compare A,C,E to B,D,F respectively. Bar = 10 µm. (TIF) [file pone.0104364.s002.tif]
